# Epigallocatechin-3-gallate Mo nanoparticles (EGM NPs) efficiently treat liver injury by strongly reducing oxidative stress, inflammation and endoplasmic reticulum stress

**DOI:** 10.3389/fphar.2022.1039558

**Published:** 2022-10-07

**Authors:** Yunrong Yang, Min Liu, Tianjiao Zhao, Qiaohui Chen, Yuqi Yang, Shuya Wang, Jinping Zhang, Guiming Deng, Kewei Sun, Yayun Nan, Ke Cao, Kelong Ai, Qiong Huang

**Affiliations:** ^1^ Department of Pharmacy, Xiangya Hospital, Central South University, Changsha, China; ^2^ National Clinical Research Center for Geriatric Disorders, Xiangya Hospital, Central South University, Changsha, China; ^3^ Xiangya School of Pharmaceutical Sciences, Central South University, Changsha, China; ^4^ Hunan Provincial Key Laboratory of Cardiovascular Research, Xiangya School of Pharmaceutical Sciences, Central South University, Changsha, China; ^5^ Department of Infection and Liver Disease, The First Hospital of Hunan University of Chinese Medicine, Changsha, China; ^6^ Geriatric Medical Center, People’s Hospital of Ningxia Hui Autonomous Region, Yinchuan, China; ^7^ Department of Oncology, The Third Xiangya Hospital, Central South University, Changsha, China

**Keywords:** epigallocatechin-3-gallate Mo nanoparticles, reactive oxygen species, acetaminophen, drug-induced liver injury, anti-inflammatory, endoplasmic reticulum stress

## Abstract

Drug-induced liver injury (DILI) is a serious clinical disease associated with reactive oxygen species (ROS) burst and subsequent inflammatory responses. However, traditional treatments were limited by low efficacy and serious side effects due to the special liver structure. Here, we developed a molybdenum (Mo)-based nanoparticles, EGM NPs, after overall consideration of the pathophysiology of DILI and the advantages of nanodrugs. It demonstrated that EGM NPs treated acetaminophen (APAP)-induced DILI by scavenging ROS and inhibiting inflammation. EGM NPs effectively scavenged various ROS and reduced cell apoptosis at the cellular level. More importantly, EGM NPs can treat APAP-induced DILI *in vivo*, reducing the levels of liver function indicators in mice with liver injury, scaling down the area of hepatocyte necrosis and successfully inhibiting endoplasmic reticulum (ER) stress in the liver. EGM NPs also showed a certain anti-inflammatory effect by reducing infiltration of macrophages, decreasing pro-inflammatory factors and inhibiting the expression levels of inducible nitric oxide synthase (NOS2) and myeloperoxidase (MPO). Collectively, our findings suggest that EGM NPs-based nanotherapeutic is a novel strategy for the treatment of DILI.

## Introduction

Drug-induced liver injury (DILI) is a common type of clinical liver injury caused by various medicines, herbal and dietary supplements or other xenobiotics (European Association for the Study of the Liver et al., 2019; Hoofnagle and Bjornsson, 2019). DILI is also one of the main causes of clinical acute liver failure, which greatly affects the treatment of patients (Thomas and Lewis, 2018). Acetaminophen (APAP) is a very typical hepatotoxic drug, whose overdose directly induced liver injury and resulted in more than 50% of liver failures in some countries with wide use of APAP (Donnelly et al., 2017; Asrani et al., 2019). APAP is also widely used in the pathogenesis and drug development research of DILI due to its representative pathological processes (Du et al., 2016; Yan et al., 2018). Despite APAP-induced liver injury having gained importance as focus of DILI, available treatments are still limited. The drug withdrawal and antioxidant small-molecule drugs are the mainstay treatment modalities, but they are not conducive to managing diseases or show inadequate efficacy. N-acetyl cysteine (NAC), as the only drug approved by the FDA for APAP-induced liver injury, also has defects like a narrow therapeutic window and insufficient efficacy (Licata et al., 2022). Once progress, transplantation is the only viable option for acute liver failure. However, only a small proportion of patients are eligible for liver transplantation due to the very limited source of donors, responsible for up to 80% of mortality on transplant-ineligible patients (Stravitz and Lee, 2019; Chayanupatkul and Schiano, 2020). It is therefore urgent that safe and effective drugs are developed against DILI.

The role of reactive oxygen species (ROS) and inflammation is indeed essential for the pathogenesis of DILI, including liver injury induced by APAP (Ye et al., 2018; Cai et al., 2022). Specifically, APAP is converted into N-acetyl-p-benzoquinone imine (NAPQI), a highly reactive metabolite, by cytochrome P450 enzyme (CYPs). Once overdosed, a large amount of NAPQI will lead to the exhaustion of glutathione (GSH, an antidote for detoxifying NAPQI). NAPQI can direct interfere the electron transport chain located in the mitochondrial membrane without hindrance from GSH, resulting in excessive ROS production (Yan et al., 2018). Increased ROS can lead to mitochondrial dysfunction, where increased ROS transfer from mitochondria to cytoplasm and further induce endoplasmic reticulum (ER) stress, phosphorylation of c-Jun N-terminal kinase (JNK) and activation of B-cell lymphoma2-associated X protein (BAX). These effects further amplify oxidative stress and mitochondrial damage to induce cell death of hepatocytes including caspase-dependent apoptosis (Yan et al., 2018; Ramachandran and Jaeschke, 2020). At the same time, because hepatocytes contain a large number of mitochondria, the impact of mitochondrial ROS is very significant (Wang et al., 2010; Xiang et al., 2021). Under the blessing of ER stress, JNK activation and BAX increase, necrotic hepatocytes release ROS and damage-associated molecular patterns (DAMPs), which further activates liver Kupffer cells (KCs) (Iorga et al., 2017; Jaeschke and Ramachandran, 2020). Activated KCs will produce a large amount of pro-inflammatory factors and chemokines, by which monocytes/macrophages and neutrophils are recruited, leading to the formation of an inflammatory environment. At the same time, the activation of NADPH oxidase 2 (NOX2), inducible nitric oxide synthase (iNOS, also known as NOS2) and myeloperoxidase (MPO) in activated inflammatory cells induced by pro-inflammatory factors will lead to the release of a large amount of ROS, which further causes oxidative stress damage to hepatocytes (Boussif et al., 2016; Yan et al., 2018). Therefore, effectively controlling ROS and inflammatory response in DILI is an effective way to treat liver injury, which is also confirmed by the therapeutic effects of some anti-inflammatory antioxidants in DILI.

Current anti-inflammatory and antioxidant drugs, however, are limited in therapeutic effect and sometimes worsen the condition of the liver (European Association for the Study of the Liver et al., 2019). The liver only accounts for 2.5% of the total body weight but receives about a quarter of the entire cardiac output, where conventional therapeutic drugs are only expose to hepatocytes for a short time and rapidly metabolized and inactivated (Greenway and Stark, 1971; Chirinos et al., 2019). In addition, small-molecule drugs enter and pass through cells primarily by passive diffusion, with little selectivity in their distribution, resulting in a variety of side effects (Madden et al., 2021; Vargason et al., 2021). The latest progress of nanomedicine has brought dawn to the treatment of DILI and antioxidant nanoparticles have also achieved excellent results as a therapeutic agent for various diseases (Sui et al., 2020; Chen et al., 2021; Zhao et al., 2022; Zhu et al., 2022; Huang et al., 2023). For example, Prussian blue (PB) is an FDA-approved antioxidant with excellent biosafety for ROS-related diseases, and a recent study reported the good therapeutic effect of PB nanoparticles on anthracycline-induced DILI (Bai et al., 2021; Gumerova and Rompel, 2021). More importantly, nanomedicines also have unique advantages to treat DILI(Liu et al., 2022): 1) 30–99% of nanoparticles tend to accumulate in the liver after entering the body through different routes; 2) nanoparticles with reasonable size can directly pass through the fenestrations (50–180 nm) of liver sinusoid and reach the lesion sites; 3) nanoparticles also have high loading capacity, targeted modification, biocompatibility and stability; 4) nanozymes have a variety of RONS scavenging abilities and exhibit more efficient antioxidant capacity. Therefore, rationally designed antioxidant nanoparticles will have very meaningful therapeutic prospects in DILI.

Recently, molybdenum (Mo) -based nanotherapeutics have shown promising therapeutic effects in the treatment of various diseases (Wang et al., 2021; Zhao. et al., 2022). As a polyvalent transition element (Mo^6+^, Mo^5+^, Mo^4+^), Mo-based nanoparticles have amazing catalytic activities due to electron transfer and valence state change, including scavenging various ROS(Liu et al., 2019). Among them, Mo-based polyoxometalate (POM) not only has unique physical and chemical properties, but also has been widely used in anti-cancer, antibacterial, anti-inflammatory and other biomedical fields due to its good biocompatibility and *in vivo* metabolic properties (Gumerova and Rompel, 2021) More importantly, the Mo atoms in POM have strong redox activity, so the well-designed Mo-based POM nanoparticles have a strong potential to eliminate ROS. By transferring charge between Mo5+and Mo6+, Mo-based nanoparticles will be capable of reducing and scavenging multiple ROS. Epigallocatechin-3-gallate (EG) is a natural polyphenolic compound obtained from green tea, has strong, where reducible polyphenol hydroxyl groups endow strong antioxidant activity (Sahadevan et al., 2022). In this study, we used EG and phosphomolybdic acid to develop Mo nanoparticles (EGM NPs) with high antioxidant activity for the treatment of DILI. Due to the very high content of Mo^5+^ratio, EGM NPs can effectively eliminate O2^·-^, ^·^OH, H_2_O_2_, ONOO^−^. To explore its therapeutic potential in DILI, we evaluated the role of EGM NPs in protecting the liver from ROS-induced injury in a mouse model of APAP-induced liver injury as well as in a cellular model. We believe that this nanoparticle with efficient ROS scavenging ability can be a valuable platform for the treatment of DILI.

## Materials and methods

### Materials

CCK8, CAT(Catalase), SOD, GSH/GSSG quantification kits were provided by Dojindo Molecular Technologies (Dojindo, Japan). APAP was purchased by Sigma-Aldrich (St. Louis, MO, USA). Malonaldehyde (MDA), alanine aminotransferase (ALT), aspartate aminotransferase (AST), total bilirubin (TBIL), blood urea nitrogen (BUN), serum creatine (SCr) test kits were supported by Nanjing Jiancheng Bioengineering Institute (Nanjing, China). Mouse NOS2, MPO, TNF-α and IL-6 ELISA kits were purchased from Elabscience Biotechnology (Houston, Texas, USA). A TUNEL assay kit (C10617). Goat anti-Rabbit IgG (H+L) Highly Cross-Adsorbed secondary antibody Alexa Fluor 488 (A11034) and MitoSOX™ Red Mitochondrial Superoxide Indicator (M36008) and ProLong™ Glass Antifade Mountant with NucBlue™ Stain (P36983) were obtained from Thermo Fisher Scientific (Carlsbad, CA, USA). Antibodies against F4/80 (ab16911), CD31 (ab9498), BAX (ab32503) and Goat Anti-Mouse IgG H&L (Alexa Fluor^®^ 594) (ab150116) were purchased fromAbcam (Cambridge, MA, USA). JNK antibody (9252S) and p-JNK antibody (4668S) were purchased from Cell Signaling Technology (Danvers, MA, USA). BCL-2 antibody (BF9103), Goat Anti-Rabbit IgG (H+L) HRP (S0001), Goat Anti-Mouse IgG (H+L) HRP (S0002) and were obtained from Affinity Bioscience (Jiangsu, China). β-actin antibody (AF5003), hydrogen peroxide assay kit (S0038) and Hoechst 33,342 Staining Solution were purchased from Beyotime Biotechnology (Shanghai, China).

### Preparation and characterization of EGM NPs

We dissolved epigallocatechin gallate (1.3 g) and phosphomolybdic acid (0.72 g) in ultra-pure water to undergo a redox reaction in an alkaline environment constructed with anhydrous sodium carbonate (3.75 g). The reaction was carried out at room temperature (RT) for 12 h, reducing the phosphomolybdic acid to a dark green solution. After that, unreacted impurities were removed by dialysis and freeze-dried to obtain EGM NPs. The shape of EGM NPs was imaged using TEM (TECNAI G2). XPS (VG ESCALAB MKII) was used to analyze the elemental valence state of EGM NPs. The UV-vis absorption spectra were collected using A580 dual beam UV/Vis spectrophotometer.

### Superoxide anion scavenging with EGM NPs

The ability of EGM NPs to scavenge O_2_^−·^ was detected by the nitro-blue tetrazolium (NBT) method. Different concentrations of EGM NPs (0.25, 0.5, 1, 2, 4 μg/ml) were mixed with methionine (20 μM), riboflavin (0.01M), NBT (0.01M), PBS (0.01M, pH7.4) and deionized water. Then, it was exposed to UV light for 5 min. Finally, the O_2_^−·^ scavenging capacity of EGM NPs was measured by EGM NPs-inhibited NBT photochemical reduction.

### Free radical scavenging with EGM NPs

The fluorescence spectrophotometry was performed to determine the OH^−.^ scavenging efficiency of EGM NPs. Different concentrations of EGM NPs (25, 50, 100, 200, 400 ng/ml) were mixed with working solution including ferrous sulfate (0.05 mM), H_2_O_2_ (1 mM), terephthalic acid (0.1 mM) and PBS (0.01M, PH7.4) and incubated for 6 min. The corresponding absorbance at 320 nm was scanned.

The UV/Vis spectrophotometry was performed to determine the H_2_O_2_ scavenging capacity of EGM NPs. First, EGM NPs (0.3 mg/ml) and H_2_O_2_ with different concentration (1.25, 2.5, 5, 10, 20 mM) were mixed, and then incubated in the dark for 12 h. The clearance rate of H_2_O_2_ was determined by detecting the ultraviolet absorption at 425 nm.

The UV-Vis spectroscopy was used to determine the scavenging ability of EGM NPs (pyrogallol red as the indicator). In brief, EGM NPs (0.4 mg/ml) and ONOO^−^ with different concentrations (0.5, 1, 2, 4 μM) were mixed and stand for 15 min. Then, the clearance rate of ONOO^−^ was measured by detecting the ultraviolet absorption.

### Cell culture and viability assay

The L02 cells were maintained with RPMI Medium 1,640 basic with 10% FBS at 37°C with 5% CO_2_. L02 cells were planted into a 96-well plate with 2 × 10^4^ cells/well. About 24 h after seeding, cells were treated with different concentrations EGM NPs with or without APAP for another 24 h or 48 h. Cell viability were tested using CCK-8 reagent at 10 μl per well for 2 h incubation at 37°C. The optical density (OD) value at 450 nm was detected using a microplate reader.

### Intracellular ROS measurement

Different concentration of EGM NPs (1, 2, 5, 10 μg/ml, respectively) with APAP (10 mM) exposed to cells for 24 h, and 50 μM of DCFH-DA ROS probe was added to wells after different treatments. Then all of them were assayed using the fluorescence microscope according to the manufacturer’s protocol. Besides, Different concentrations of EGM NPs (1, 2, 5, 10 μg/ml, respectively) with APAP (10 mM) exposed to cells for 24 h. After that, these cells were collected and grounded in extraction solution on ice. Finally, the H_2_O_2_ content was measured using kits according to the manufacturer’s instructions.

### Mitochondrial function

Living L02 cells after different treatments were labeled with 5 μM MitoSOX™ reagent for 10 min at 37°C followed with Hoechst 33,342 Staining Solution for Live Cells and mount in warm buffer. After that, the cells were washed for three times and examined using fluorescence microscope.

### Western blot analysis

The protein was extracted from liver tissue or L02 cells in RIPA buffer containing PMSF and phosphatase inhibitors. The protein concentration was quantified by BCA. The lysates were added with 4× Laemmlisample buffer, boiled and separated using SDS-PAGE. Then, the proteins were transferred to PVDF membrane. The membranes were blocked for 1 h at RT with 5% skimmed milk, and incubated with primary antibodies against p-JNK (dilution 1:1,000), JNK (dilution 1:1,000), BAX (dilution 1:1,000), BCL-2 (dilution 1:1,000) and β-actin (dilution 1:5,000) overnight at 4°C. After that, the membrane was washed three times with TBST, and then incubated with HRP-conjugated secondary antibodies for 1 h at RT. Finally, the membrane was treated with enhanced chemiluminescence. The protein bands were evaluated by using Image J software.

### Animal care conditions

Male ICR mice (4–6 weeks old, 18–22 g) were obtained from Hunan STA Laboratory Animal Co., Ltd. (Changsha, China). These animals were fed with a standard diet and water for 7 days in a clean environment at 24 ± 2°C and 12 h light/dark cycle. All experimental procedures were approved by the Institutional Animal Care and Use Committee (IACUC), Xiangya Hospital, Central South University, China.

### APAP-induced hepatotoxicity model and experimental design

Mice were injected intraperitoneally with APAP (250 mg/kg) after fasting for 12 h without water to induce hepatotoxicity model. Two hours after the APAP-induced hepatotoxicity model induction, the mice were randomly allocated into the following groups: APAP group, APAP+EGM NPs group (i.v., 0.5, 1.0, and 2.0 mg/kg), APAP+NAC group (i.p., 300 mg/kg). Sham group was treated with saline. All mice were euthanized 24 h after APAP administration. The blood was collected and serum was separated by 2000 g/min for 15 min. The tissues were collected and kept at -80°C.

### Biochemical assays

The levels of ALT, AST and TBIL in serum were determined using assay kits. Hepatic homogenates were used for detecting the levels of CAT, MDA, SOD and GSH/GSSH ratio following assay kits by the manufacturer’s instructions. The hepatic homogenates levels of NOS2, MPO, IL-6 and TNF-α were measured by enzyme-linked immunosorbent assay (ELISA) kits according to the manufacturer’s protocols.

### Histological analysis

Fresh liver tissues were gathered, fixed instantly with 4% paraformaldehyde, and then embedded in paraffin. Lastly, the liver tissues were cut into a thickness of 5 μm sections, which were stained with hematoxylin-eosin (H&E) staining and Masson staining to assess the liver pathological changes using a light microscopy.

### Superoxide anion assay

Liver tissue superoxide anion production was analyzed by staining with dihydroethidium (DHE). The frozen liver tissue slices were incubated with DHE for 30 min in the dark and washed with 1×PBS. Then incubate with DAPI solution at RT for 3 min and washed with 1×PBS. And the images were captured by using Ortho-Fluorescent Microscopy (Nikon ECLIPSE C1).

### Immunofluorescence

Immunofluorescence staining was used to detect the F4/80 and CD31 in the liver. The anti-F4/80 antibody (Abcam, USA, dilution 1:50) and anti-CD31 antibody (Abcam, USA, dilution 1:500) were incubated overnight at 4°C. Then exposed to Alexa Fluor-488-conjugated goat-anti-rabbit (Invitrogen, dilution 1:500) and goat anti-mouse IgG H&L (Alexa Fluor^®^ 594, Abcam, USA, dilution 1:500) for 1 h at RT. Nuclei were counterstained with ProLong™ Glass Antifade Mountant with NucBlue™ Stain. Images were acquired by using a fluorescence confocal microscope.

### TUNEL staining

Liver tissue apoptosis was measured using Transferase-mediated deoxyuridine triphosphate-biotin nick end labeling (TUNEL) staining according to the manufacture’s protocol. The sections were observed under a fluorescence microscope. The fluorescence intensity was quantified using Image J software.

### Quantitative RT-PCR analysis

Total RNA was extracted from frozen liver tissues using TRIzol and reverse-transcribed to obtain cDNA. Quantitative real-time PCR was performed with Applied Biosystems StepOnePlus instrument and TB Green Premix Ex TaqTM (Tli RNaseH Plus). Gene expression was evaluated by the comparative-Ct Method, using β-actin as a reference gene. The sequences of primers are listed in Table 1.

**TABLE 1:** Primers used for quantitative real-time PCR.

Target gene	Primer	Sequence (5′-3′)
CHOP	Forward	GTC​CCT​GCC​TTT​CAC​CTT​GG
	Reverse	GGT​TTT​TGA​TTC​TTC​CTC​TTC​G
Bip	Forward	TGT​GTG​TGA​GAC​CAG​AAC​CG
	Reverse	TAG​GTG​GTC​CCC​AAG​TCG​AT
sXBP1	Forward	CTG​AGT​CCG​AAT​CAG​GTG​CAG
	Reverse	GTC​CAT​GGG​AAG​ATG​TTC​TGG
β-actin	Forward	ACGAGGCCCAGAGCAAGA
	Reverse	TTG​GTT​ACA​ATG​CCG​TGT​TCA

### Statistical analysis

Data were expressed as Mean ± SD. Groups were compared by one-way analysis of variance (ANOVA) followed by a post hoc multiple comparison test using SPSS version 22.0. Differences with *p* < 0.05 were considered statistically significant.

## Results

### Synthesis and characterization of EGM NPs

**FIGURE 1:**
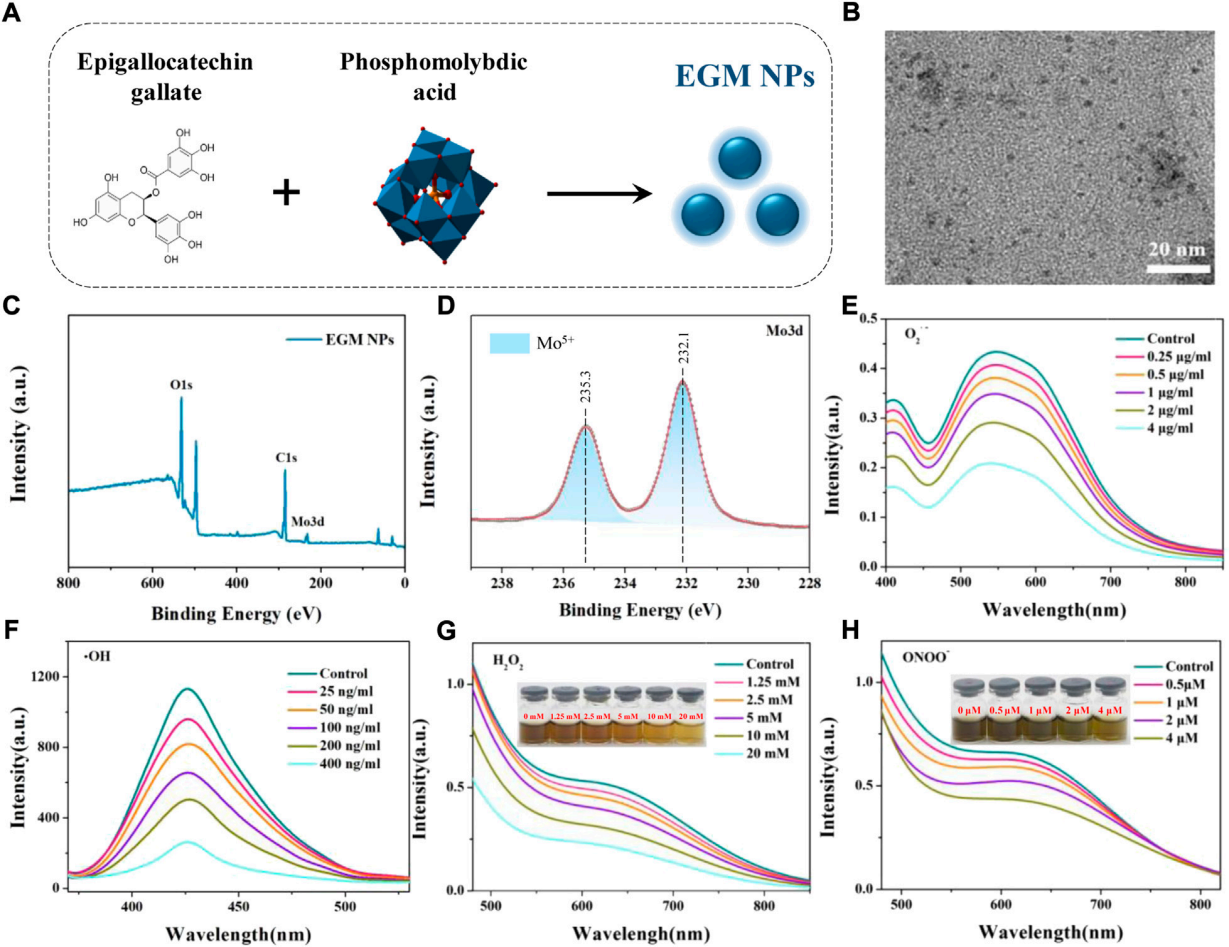
Synthesis and characterization of EGM NPs. **(A)** Schematic illustration of synthesis of EGM NPs. **(B)** TEM image of EGM NPs in water, scale bar: 20 nm. **(C)** X-ray photoelectron spectroscopy (XPS) spectrum of EMG NPs. **(D)** XPS spectrum of Mo in EMG NPs. **(E–H)** O_2_^.-^ **(E)**, OH **(F)**, H_2_O_2_ **(G)**, and ONOO^−^ **(H)** scavenging ability of EGM NPs.

In this study, EGM NPs were prepared by redox method using EG to reduce phosphomolybdic acid under alkaline conditions (Figure 1A). The TEM results showed that the EGM NPs had good dispersion of nanodots and uniform size with a diameter of 1.92 ± 0.38 nm (Figure 1B). EGs also were successfully modified to the surface of EGM NPs during the preparation process (Figure 1C). The constitute of Mo^5+^ was confirmed in EGM NPs (Figure 1D), which may responsible for strong ability to scavenge various ROS. Therefore, we examined the scavenging ability of EGM NPs for O2^·-^, H_2_O_2_, ^·^OH, ONOO^−^. EGM NPs had strong superoxide (SOD)-mimic activity to effectively scavenge O2^·-^, reaching 270 U/mg (Figure 1E). They also showed excellent catalase (CAT)-mimic activity to catalyze the scavenging of H_2_O_2_, where H_2_O_2_ was significantly eliminated after 8 h co-incubation with 20 mM EGM NPs (Figure 1F). In addition, EGM NPs can effectively scavenge OH, which is the most toxic secondary ROS produced in DILI (Figure 1G). A Pyrogallol Red bleaching assay was employed to obtain the ONOO^−^ scavenging ability. EGM NPs exhibited high ONOO^−^ scavenging activity, and ONOO^−^ was significantly quenched after incubation with 4 μM EGM NPs (Figure 1H).

### Antioxidant properties and cellular protection of EGM NPs

**FIGURE 2:**
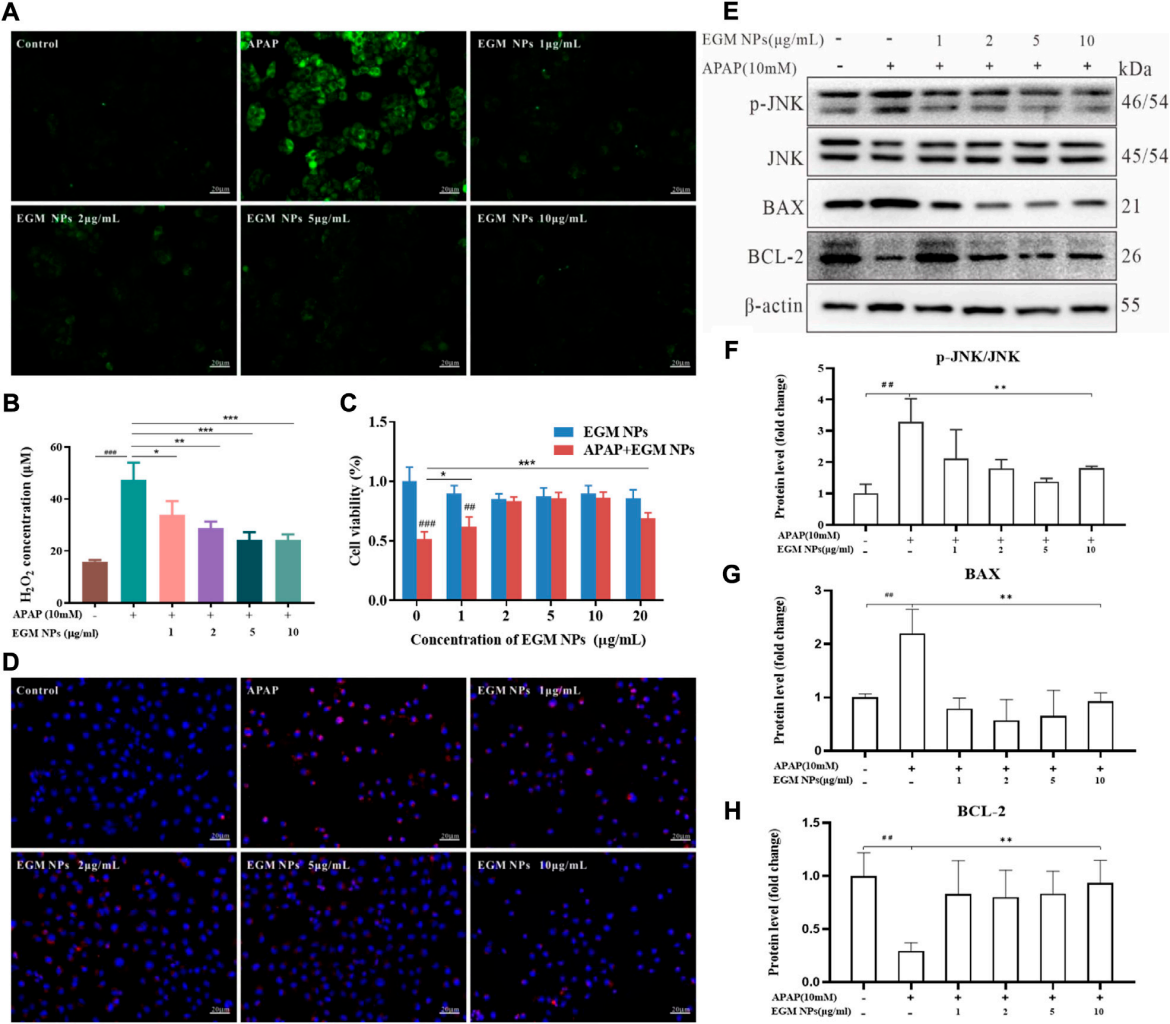
Antioxidant properties and cellular protection of EGM NPs. **(A)** Detection of ROS generation by DCFH-DA in L02 cells after different treatments. Scale bar: 20 μm. **(B)** H_2_O_2_ concentrations generated by L02 cells after different treatments. **(C)** Cell viability of L02 cells after different treatments. Data represent means ± S.D. from six independent replicates. **(D)** Fluorescence images of MitoSOX staining in L02 cells after different treatments. Scale bar: 20 μm. **(E)** Western blot analysis of p-JNK, JNK, BAX and BCL-2 proteins expression in L02 cells after different treatments. **(F–H)** Quantification of the protein immunoblots of p-JNK/JNK **(F)**, BAX **(G)** and BCL-2 **(H)**. Data represent means ± S.D. from three independent replicates. (^**^ *p* < 0.01vs*.* APAP group; ^##^ *p* < 0.01 vs*.* Control group).

The role of oxidative stress caused by the hepatotoxic drugs-induced ROS burst in the development of DILI is extremely striking and critical (Du et al., 2016; Yan et al., 2018). We mimicked APAP-induced hepatocyte injury *in vitro* by incubating L02 cells with APAP and evaluated the cytoprotective effect of EGM NPs. DCFH-DA is a non-fluorescent probe that can be hydrolyzed to DCFH, which can subsequently be converted to fluorescent DCFH by ROS. As shown in Figure 2A, strong green fluorescence was observed after APAP stimulation, and the fluorescence intensity was significantly attenuated in L02 cells treated with EGM NPs. In all, ROS was significantly increased in L02 cells after the treatment of 10 mM APAP for 24 h by measuring H_2_O_2_ in different treatment groups, but was significantly decreased after EGM NPs treatment (Figure 2B). To validate the anti-oxidative stress efficacy of EGM NPs, we evaluated their cytoprotective effect by the CCK-8 assay. As indicated in Figure 2C, EGM NPs significantly ameliorated the decrease of cell viability caused by APAP. Taken together, APAP significantly induced ROS production and decreased cell viability in L02 cells, while EGM NPs exerted antioxidant effects to reduce ROS level and protect hepatocytes from APAP-induced injury.

NAPQI, the active metabolite of APAP in the liver, can directly interfere with mitochondrial ETC, resulting in the production of a large amount of mitochondrial ROS. After mitochondrial ROS is transferred to the cytoplasm, it activates JNK and regulates apoptosis-related proteins of B-cell lymphoma2 (BCL) family, thereby amplifying mitochondrial damage and leading to apoptosis (Wang, 2014; Iorga et al., 2017). Therefore, a mitochondrial ROS-specific fluorescent probe (MitoSOX) was employed to explore the relevant changes in mitochondrial ROS in L02 cells. As shown in Figure 2D, APAP induced an increase in mitochondrial ROS levels of L02 cells. L02 cells co-incubated with APAP and EGM NPs showed weak red fluorescence, indicating that EGM NPs effectively inhibited the accumulation of mitochondrial ROS. Furthermore, we found that APAP stimulated the activation of the JNK signaling pathway, increasing the phosphorylation of JNK. At the same time, APAP also led to a significant up-regulation of the apoptotic protein BAX, accompanied by a decrease in the anti-apoptotic protein BCL-2. After EGM NPs treatment, the activation of JNK was inhibited, while BAX was downregulated and BCL-2 was upregulated (Figures 2E–H). It indicated that EGM NPs effectively reduced cell apoptosis by reducing the level of mitochondrial ROS.

### Therapeutic effects of EGM NPs on APAP-induced liver injury

**FIGURE 3:**
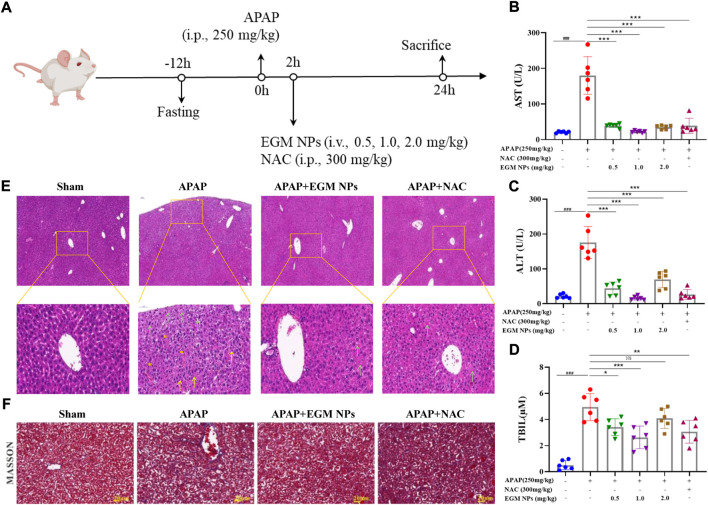
Therapeutic effects of EGM NPs on APAP-induced liver injury. **(A)** Schematic illustration of the establishment and treatment schedule of APAP-induced liver injury mice. Serum levels of ALT **(B)**, AST **(C)** and TBIL **(D)** in normal mice or APAP-induced liver injury mice at 24 h after different treatments. **(E)** HE staining of liver tissues and their enlarged images from each group. **(F)** Masson-trichrome staining of liver tissues from each group. Scale bar: 20 μm. Data represent means ± S.D. from six independent replicates. (^*^ *p* < 0.05, ^**^ *p* < 0.01, ^***^ *p* < 0.001 vs*.* APAP group; ^###^ *p* < 0.001 vs*.* Sham group).

Considering the role of EGM NPs in mitigating APAP hepatotoxicity *in vitro*, we further explored their therapeutic effects on an *in vivo* model of APAP-induced liver injury. We established a DILI mice model by intraperitoneal injection (i.p.) of 250 mg/kg APAP, and NAC was used as a positive control (Figure 3A). Different treatment modalities were performed 2 h after APAP injection: saline, EGM NPs [0.5, 1.0, 2.0 mg/kg, tail vein injection (i.v.)], NAC [300 mg/kg, intraperitoneal (i.p.)]. Finally, the mice were sacrificed 24 h after treatment administration, and the corresponding examinations were carried out. ALT, AST, and TBIL are important biochemical indicators of liver function. As shown in Figures 3B–D, mice treated with APAP displayed significantly high levels of AST, ALT, and TBIL, indicating DILI mice model was successfully constructed. The low, middle and high doses of EGM NPs all significantly reduced AST, ALT, and TBIL in DILI mice, and the effects were comparable to the positive drugs, which demonstrated the protective effect of EGM NPs in DILI mice. The middle dose of EGM NPs (1.0 mg/kg) showed the best effect, we used this dose in the following experiment.

We further observed histopathological changes in liver tissue during DILI. HE staining showed that liver injury in DILI mice was characterized by hepatocyte necrosis, formation vacuolar degeneration around cental veins, irregular arrangement of hepatocytes, and inflammatory cell infiltration (Figure 3E). These histological abnormalities were partially abolished by treatment with EGM NPs. Masson staining showed that there were obvious collagen deposition and necrotic lesions in the liver tissue of DILI mice, and the treatment of EGM NPs also improved the necrosis of the liver tissue in mice models (Figure 3F).

### EGM NPs scavenge ROS to alleviate ER stress and cell apoptosis

**FIGURE 4:**
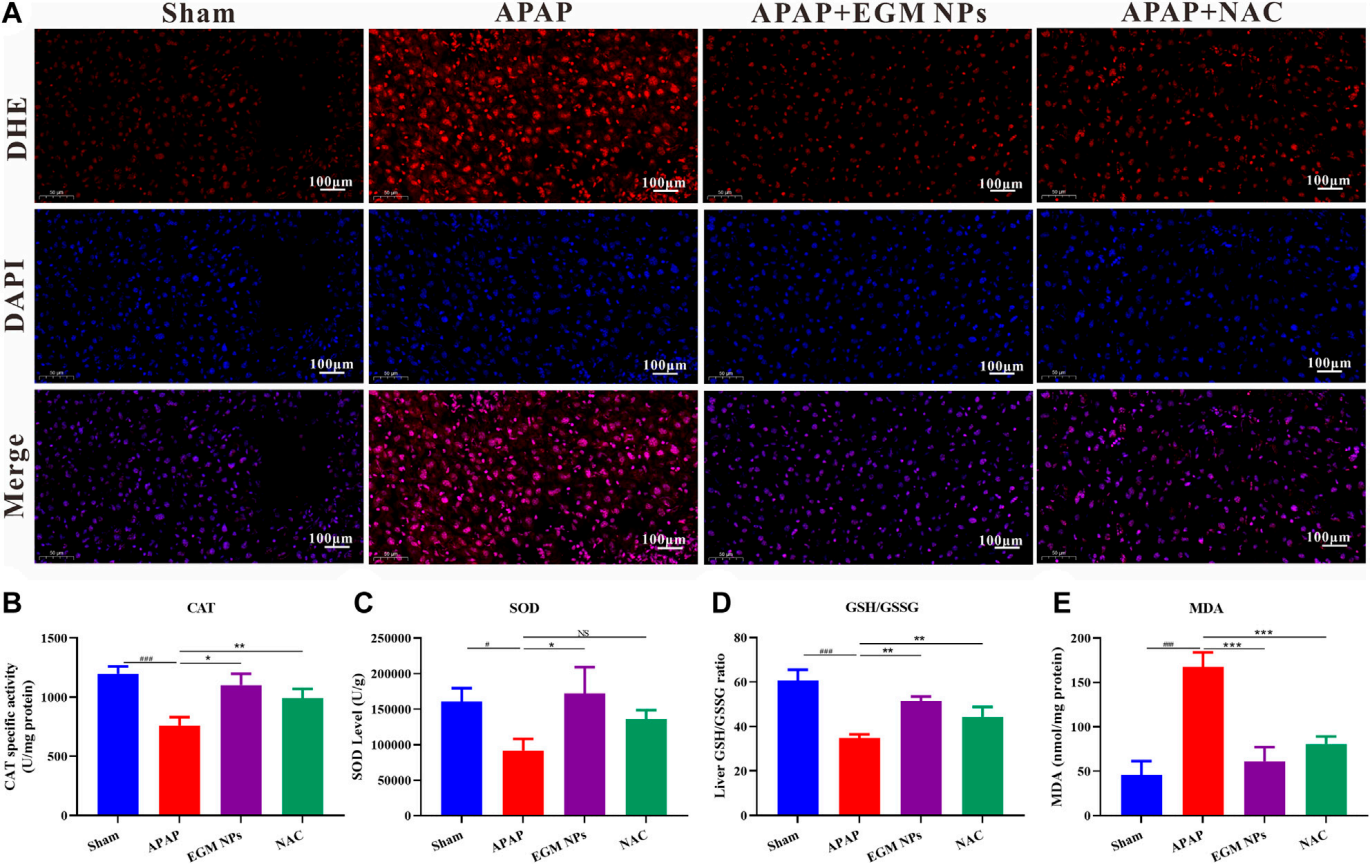
EGM NPs scavenge ROS to alleviate APAP-induced liver injury. **(A)** DHE staining (red fluorescence), DAPI (blue fluorescence) staining, and their merge images of liver tissues from each group. Scale bar: 100 μm. **(B–E)** CAT **(B)**, SOD **(C)**, GSH/GSSG **(D)**, and MDA **(E)** levels measured in liver tissue homogenates from each group. Data represent means ± S.D. from three independent replicates. (*p<0.05, **p<0.01, ***p<0.001 vs. APAP group; ^#^ *P*<0.5, ^###^ *P*<0.001 vs. Sham group).

We have demonstrated the strong antioxidant capacity of EGM NPs *in vitro* and the excellent DILI therapeutic effect *in vivo*. Therefore, we further explored the therapeutic mechanism *in vivo*. DHE is a widely used redox-sensitive fluorescent probe that is specific to ROS such as O2^·-^ and H_2_O_2_. As shown in Figure 4A, strong red fluorescence of DHE was displayed in the liver of DILI model mice, while EGM NPs significantly reduced the fluorescence signal intensity. The alternations in antioxidant enzyme activity (CAT activity, SOD activity), redox state (GSH/GSSG), and oxidative damage (MDA, a lipid peroxidation product) in liver tissue further validated the efficacy of EGM NPs as ROS scavengers (Figures 4B–E). Decreased CAT and SOD enzyme activities, decreased ration of GSH/GSSG and increased MDA were observed in APAP-treated mice, which indicated that the redox state of the liver shifted toward oxidation and oxidative stress were aggregated. Compared with the DILI model group, EGM NPs showed efficacy in reversing these indicators, which further confirmed the antioxidant effect of EGM NPs in DILI.

**FIGURE 5:**
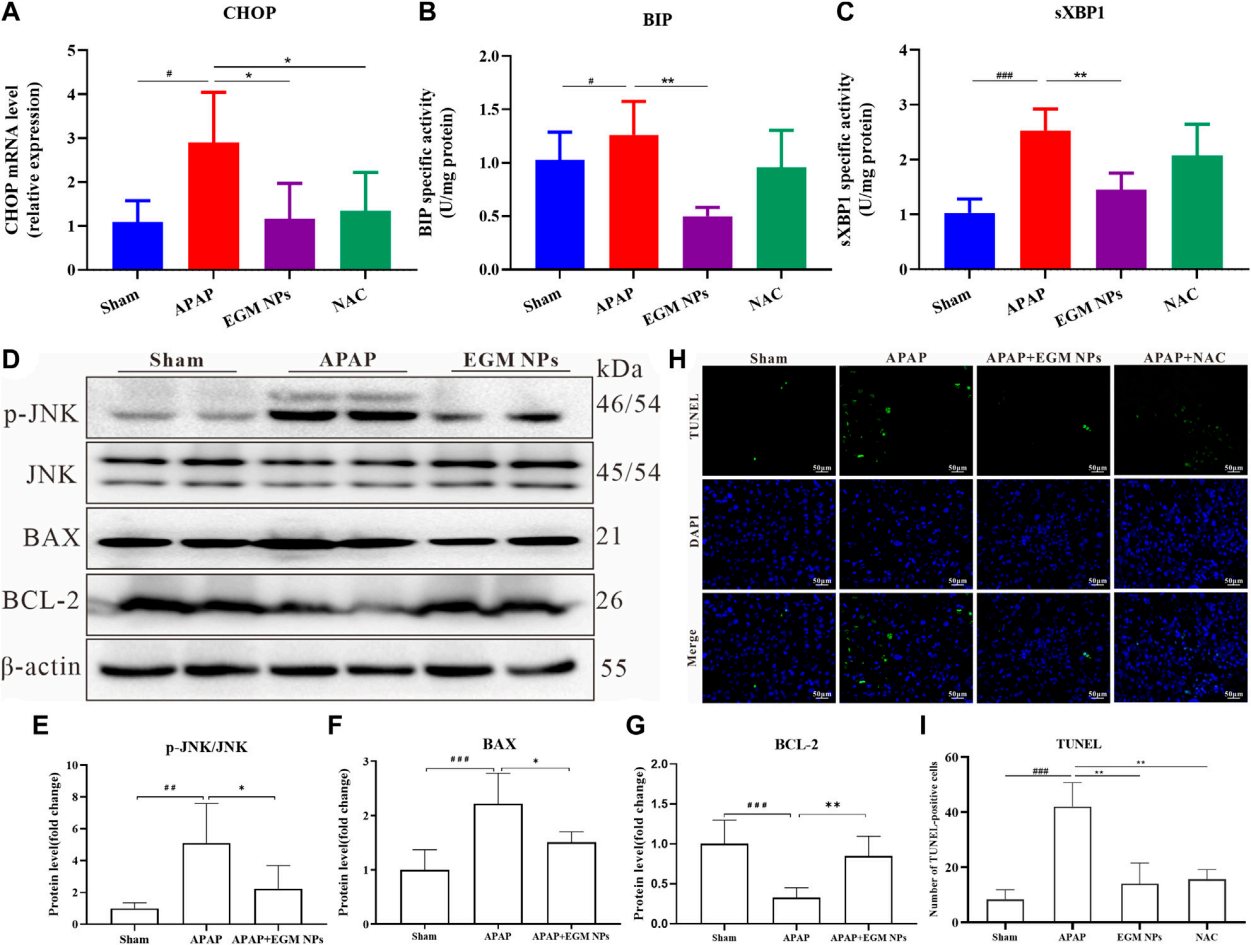
EGM NPs alleviate ER stress and cell apoptosis. **(A–C)** mRNA levels of CHOP **(A)**, BIP **(B)** and sXBP1 **(C)** in liver tissue homogenates from each group. **(D)** Western blot analysis of p-JNK, JNK, BAX and BCL-2 proteins expression in liver tissue homogenates from each group. **(E,F)** Quantification of the protein immunoblots of p-JNK/JNK **(E)**, BAX **(F)** and BCL-2 **(G)**. TUNEL staining (green fluorescence), DAPI (blue fluorescence) staining, and their merge images of liver tissues from each group. Scale bar: 50 μm. **(I)** Quantification of TUNEL-positive cells in **(H)**. (^*^ *p* < 0.05, ^**^ *p* < 0.01, vs*.* APAP group; ^##^ *p* < 0.01, ^###^ *p* < 0.001 vs*.* Sham group).

Oxidative stress will result in inefficient protein folding and increased levels of misfolded proteins, which in turn induces severe ER stress. Bip/GRP78, CHOP and spliced XBP1 (sXBP1) are marker genes of ER stress, which are up-regulated during ER stress (Bhattarai et al., 2021). Therefore, we detected the expression levels of Bip, CHOP and sXBP1 to evaluate ER stress. The qRT-PCR results showed that Bip, CHOP and sXBP1 mRNA were upregulated in liver tissues after APAP treatment. Treatment with EGM NPs significantly decreased the gene expression of Bip, CHOP and sXBP1 as compared with the APAP group (Figures 5A–C). Similarly, we also examined the effect of EGM NPs on APAP-induced cell apoptosis *in vivo*. In DILI mice treated with EGM NPs, the phosphorylation level of JNK was significantly decreased, the expression of BAX was significantly decreased, and the level of BCL-2 was increased (Figures 5D–G). The apoptosis of liver cells was further verified by TUNEL staining. As shown in Figures 5H,I, EGM NPs significantly reduced apoptosis in the liver region of DILI mice. Therefore, EGM NPs can alleviate ER stress and cell apoptosis in the liver by scavenging ROS, thereby alleviating APAP-induced liver injury.

### EGM NPs inhibit inflammation during APAP-induced liver injury

**FIGURE 6:**
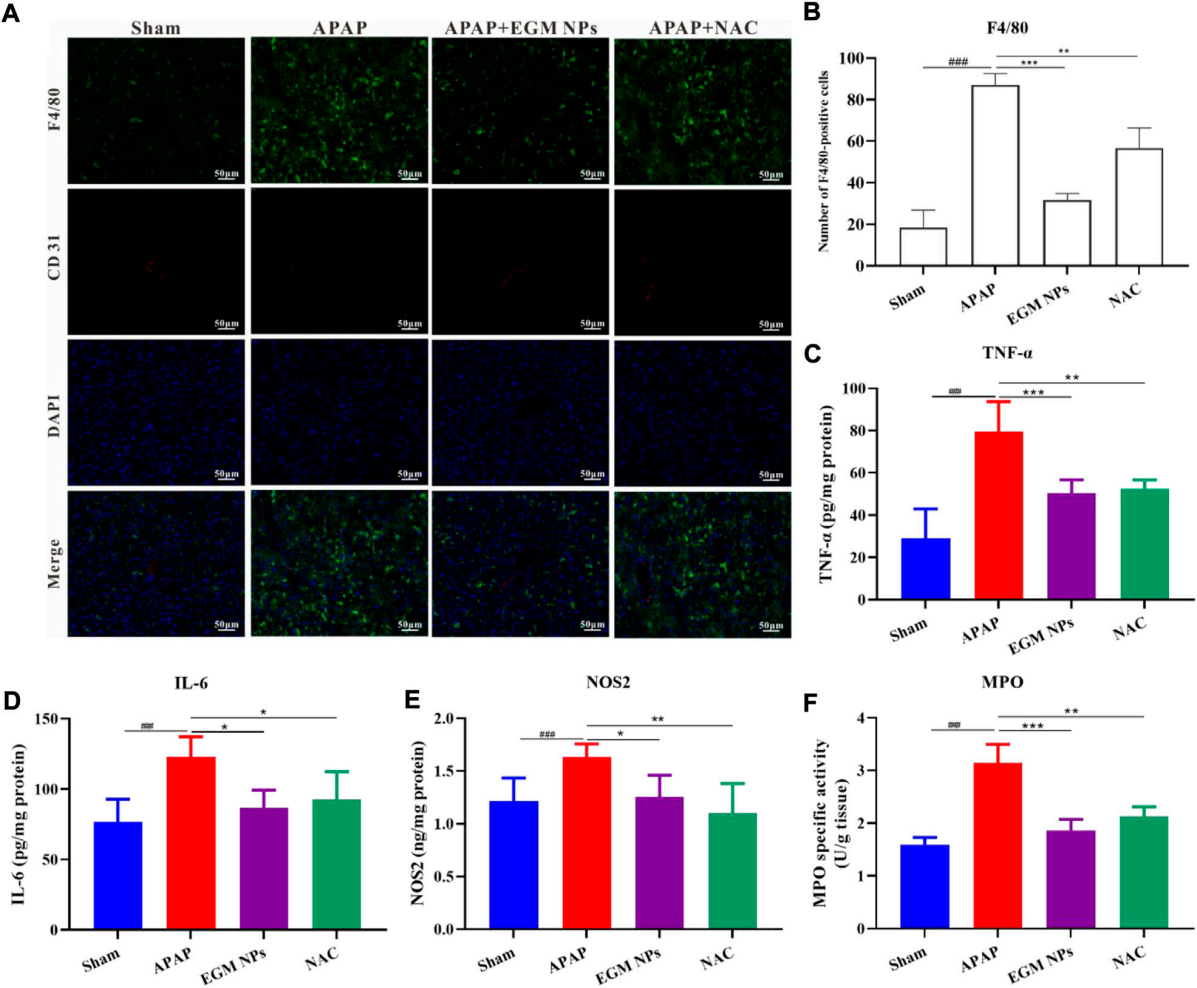
EGM NPs inhibit inflammation during APAP-induced liver injury. **(A)** F4/80 staining (green fluorescence), CD31 (red fluorescence) staining, DAPI (blue fluorescence) staining, and their merge images of liver tissues from each group. Scale bar: 50 μm. **(B)** Quantification of F4/80-positive cells in **(A)**. Data represent means ± S.D. from three independent replicates. **(C–F)** TNF-α **(C)**, IL-6 **(D)**, NOS2 **(E)**, and MPO **(F)** levels measured in liver tissue homogenates from each group. Data represent means ± S.D. from six independent replicates. (^*^ *p* < 0.05, ^**^ *p* < 0.01, ^***^ *p* < 0.001 vs*.* APAP group; ^###^ *p* < 0.001 vs*.* Sham group).

APAP-induced ROS can lead to the release of various DAMPs by inducing hepatocyte death during the development of DILI, thereby recruiting monocytes/macrophages and neutrophils. A large number of inflammatory factors and ROS are generated and a vicious cycle of inflammation and ROS is formed, which exacerbates APAP-induced liver injury (Yan et al., 2018; Cai et al., 2022). Therefore, the effect of EGM NPs on inflammatory response is also worthy of further study. By macrophage-specific F4/80 staining, it was found that macrophage infiltration in the liver tissue of DILI mice was significantly increased compared with the sham group (Figures 6A,B). And EGM NPs reduced macrophage infiltration in DILI. In addition, EGM NPs also significantly reduced the levels of the proinflammatory factors TNF-α and IL-6 in DILI (Figures 6C,D). NOS2 is significantly up-regulated after activation of inflammatory cell, and MPO is also significantly increased when neutrophils are recruited to inflamed sites. The up-regulation of NOS2 and MPO in inflammatory cells also leads to the production and release of ROS, aggravating the oxidative stress damage on liver. As expected, NOS2 and MPO levels were also significantly downregulated in EGM NPs-treated DILI mice (Figures 6E,F). Therefore, EGM NPs had good anti-inflammatory and antioxidant effects, thereby reducing the inflammatory response in DILI mice.

### Biocompatibility assessment of EGM NPs

**FIGURE 7:**
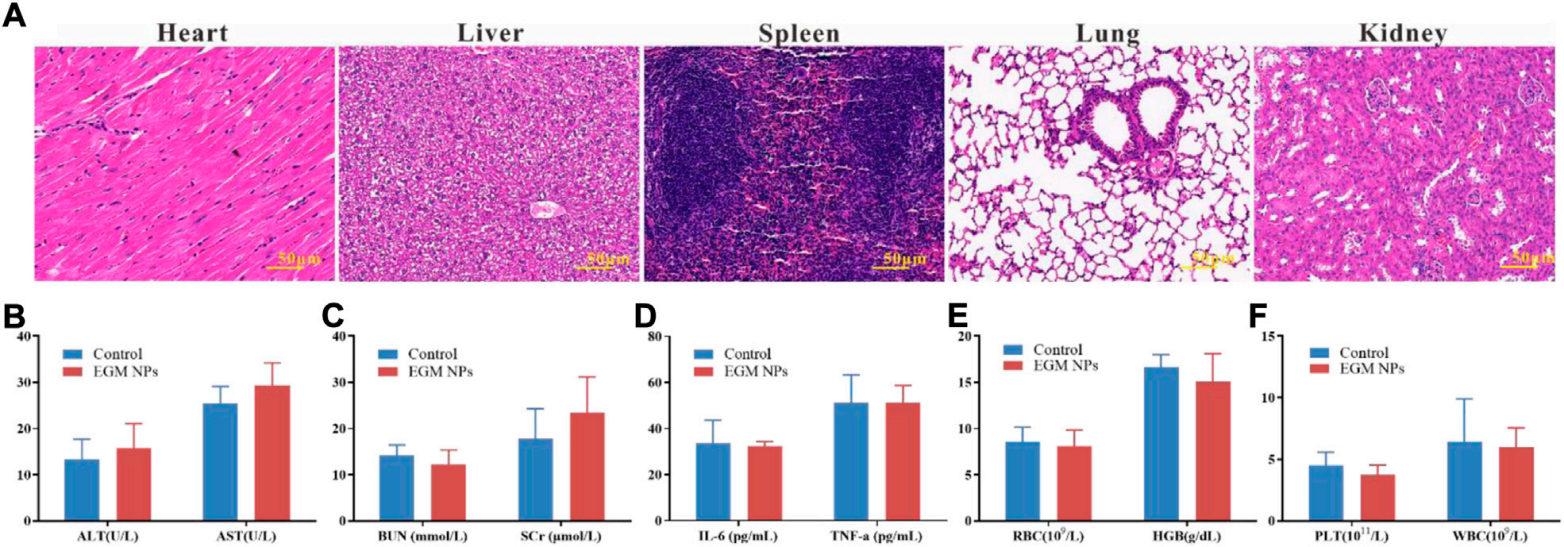
Biocompatibility assessment of EGM NPs. **(A)** HE staining of major organs (heart, liver, spleen, lung, kidney) in normal mice 24 h after intravenously injection with EGM NPs. **(B–D)** Serum levels of liver function indicators **(B)**, kidney function indicators **(C)** and inflammatory factors **(D)** in normal mice (control group), and mice intravenously injected with EGM NPs, 24 h after injection. **(E,F)** Blood parameters and in normal mice (control group), and mice injected with EGM NPs, 24 h after injection. Data represent means ± S.D. from six independent replicates.

The safety of nanomedicines always remains the highest priority, which is of great significance for future clinical translation (Huang et al., 2022; Xiao et al., 2022; Yang et al., 2022). The results of the CCK-8 assay showed that the cell viability was not inhibited significantly when L02 cells were exposed to relatively high concentrations of EGM NPs for a long time (Supplementary Figure S1). Therefore, EGM NPs had good biocompatibility to some extent. In addition, major organs (heart, liver, spleen, lung, and kidney) had no obvious pathological damage (Figure 7A), and EGM NPs did not affect liver function (AST, ALT), renal function (BUN, SCr), levels of inflammatory factors (IL-6, TNF-α) and blood route parameters (RBC, HGB, PLT, WBC) in normal mice (Figures 7B–F). Even after 1 month of treatment with the highest dose (32 mg/kg) of EGM NPs, major organs such as heart, liver, spleen, lung, and kidney showed no obvious pathological damage (Supplementary Figure S2).

## Discussion

DILI is one of the leading causes of hospitalization and drug withdrawal from the market, which can cause severe liver injury and even acute liver failure, resulting in a huge global disease burden. The susceptibility to DILI is also influenced by various factors, especially in the elderly (Lucena et al., 2020). The incidence of serious adverse drug reactions also increased with age. Physiological changes and increased individual variability, along with a variety of diseases and frailty syndromes, may increase susceptibility to DILI in the elderly. For example, the incidence of APAP overdose accounts for only 15% of cases in the general population, but 55% of cases in the elderly (Mitchell and Hilmer, 2010). At the same time, the incidence of DILI in elderly patients and the difficulty of treatment are also increased due to the problem of comorbid drug use. The clinical treatment of DILI is very limited, and there is an urgent need for effective and safe drugs. The burst of ROS is believed to have a key role in the pathogenesis of DILI, and thus antioxidants are considered potentially effective therapeutic strategies, especially those with broad and strong ROS scavenging effects. Therefore, in this study, small-sized antioxidant nanoparticles, EGM NPs, were used to treat APAP-induced liver injury.

Considering the representativeness of APAP in DILI, this study mainly adopted the animal model of APAP-induced DILI. At normal therapeutic doses, APAP is mostly metabolized and converted into nontoxic compounds while 5–9% of APAP is converted into NAPQI under the action of CYP2E1. NAPQI is detoxicated by GSH and does not cause toxicity to liver cells. When overdosed, the original metabolic enzymes will be saturated and a large number of NAPQI will be produced, which will lead to GSH depletion (Yan et al., 2018). NAPQI directly interferes with ETC, resulting in the massive production of ROS. Mitochondria are the major source of intracellular ROS and play a key role in the production of ATP, the regulation of various catabolic and anabolic processes, and the maintenance of redox homeostasis (Bonora et al., 2022; Marchi et al., 2022). Under normal physiological conditions, ETC produces ROS as a by-product during oxidative phosphorylation process. Once ETC disrupted, electrons leak to form high levels of O2^·-^. And H_2_O_2_ is generated under the action of mitochondrial SOD, which is further converted to OH through the Fenton reaction. At the same time, O2^·−^may react with NO under the catalyze of NOS2 to form ONOO^−^ and hypochlorous acid (HOCl) are formed under the action of MPO (Liu et al., 2022). Traditional antioxidants tend to scavenge only one type of ROS, so the antioxidant effect is very limited. In this study, EGM NPs can not only scavenge O2^·-^, but also showed strong scavenging ability for OH, H_2_O_2_, ONOO^−^. EGM NPs also reduced liver NOS2 and MPO in DILI mice, which proved that EGM NPs exert an enormous function on reducing ROS accumulation in DILI.

**FIGURE 8:**
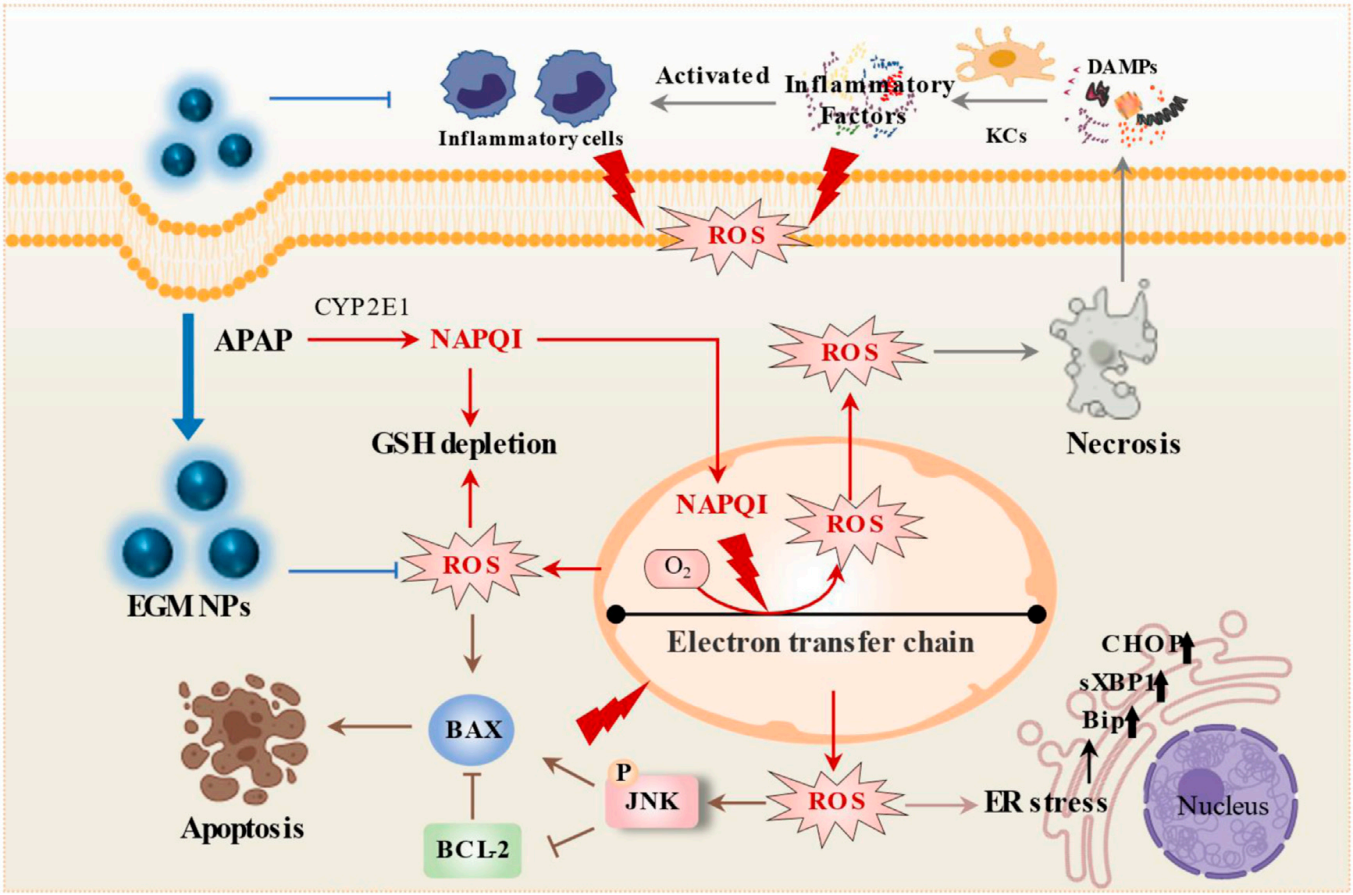
Schematic illustration of therapeutic mechanism of TWNDs for APAP-induced liver injury.

As highly reactive species, ROS can cause oxidative damage to mitochondrial proteins, lipids, and nucleic acids, which in turn leads to mitochondrial dysfunction and triggers a series of signaling cascades to cell death. Intracellular ROS, which disrupt ER function, induce ER stress based on the accumulation of unfolded and misfolded proteins in the ER. Unfolded and misfolded proteins in the ER lead to the release of Bip and activation of ER transmembrane protein (IRE1α, PERK and ATF6) (Xiang et al., 2022). The phosphorylated IRE1α will covert e unspliced XBP1 protein (uXBP1) to the highly active sXBP1. And when the ER continues to aggravate, ATF4 initiates the transcription of CHOP, which initiates and promotes cell apoptosis. In addition, ROS also activate JNK through the MAP3 kinase (MLK2, ASK-1)-MKK4/7 (MAP2 KINASE) pathway and the GSK-3β pathway (Levada et al., 2018; Lee et al., 2019). p-JNK will be transported to the mitochondria and then interferes with ETC, generates ROS, and forms an injury cycle. And the effects of oxidative stress are amplified. Cytoplasmic ROS also triggers increased expression of BAX, and JNK activation affects the BCL family. BAX migrates to the mitochondrial membrane, damages the mitochondrial membrane, and induces caspase-dependent apoptosis, while BCL-2 can inhibit this process (Weston and Davis, 2007; Yan et al., 2018; Ramachandran and Jaeschke, 2020). This study found that treatment with EGM NPs can effectively reduce ROS level *in vitro* and *in vivo*. And they can alleviate ER stress with decreased level of Bip, sXBP1 and CHOP and reduce cell apoptosis with inhibition of p-JNK, downregulation of BAX, and upregulation of BCL-2 (Figure 8). Inflammation is also an important factor in DILI. DAMPs, pro-inflammatory factors (IL-6, TNF-α, etc.) and chemokines activate and recruit various inflammatory cells to exert innate immunity. Notably, ROS can also directly activate inflammatory cells. Activated inflammatory cells induce apoptosis by releasing cytokines such as TNF-α and FasL (Yan et al., 2018; Kong et al., 2019). The upregulation of NOX2, NOS2 and MPO in inflammatory cells bring a large amount of ROS, causing bystander injury and leading to hepatocyte death (Liu et al., 2022). In this study, it was found that EGM NPs inhibited inflammatory cell infiltration in DILI, decreased the levels of pro-inflammatory factors IL-6 and TNF-α, and inhibited the expression of NOS2 and MPO in addition to effectively scavenging ROS in the liver (Figure 8). Finally, this study also confirmed the biocompatibility of EGM NPs *in vitro* and *in vivo*.

Taken together, we confirmed that EGM NPs ameliorated APAP-induced DILI by effectively scavenging ROS and inhibiting the inflammatory response, suggesting that EGM NPs have great therapeutic potential in DILI. To our knowledge, it is the first use of Mo-based POM to treat DILI, which has important implications for the effective treatment and prevention of DILI in the future.

## Data Availability

The original contributions presented in the study are included in the article/Supplementary Material, further inquiries can be directed to the corresponding author.

## Supplementary material

Supplementary Figure S1 In vitro cytotoxicity of EGM NPs towards L02 cells after incubation for 24 h and 48 h. Data represent means ± S.D. from six independent replicates.

Supplementary Figure S2 HE staining of major organs (heart, liver, spleen, lung, kidney) of normal mice after injection with PBS or EGM NPs for 30 days.
