# Supplementary figures and images for: Epigallocatechin-3-gallate Mo nanoparticles (EGM NPs) efficiently treat liver injury by strongly reducing oxidative stress, inflammation and endoplasmic reticulum stress
Source: Front Pharmacol. 2022 Oct 7;13:1039558. doi: 10.3389/fphar.2022.1039558 (PMC9585210; doi:10.3389/fphar.2022.1039558)

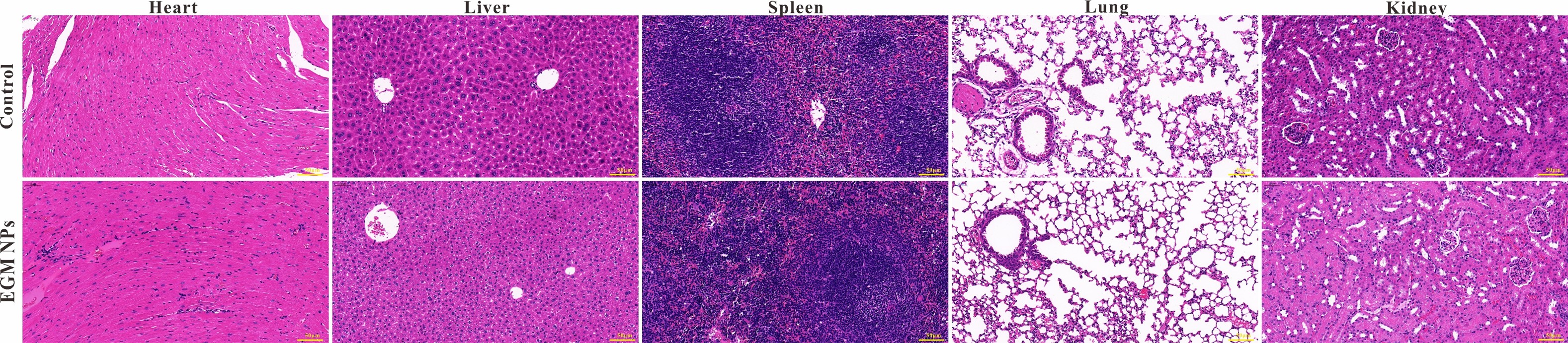

Supplement: Supplementary file 1 [file Image2.jpg]

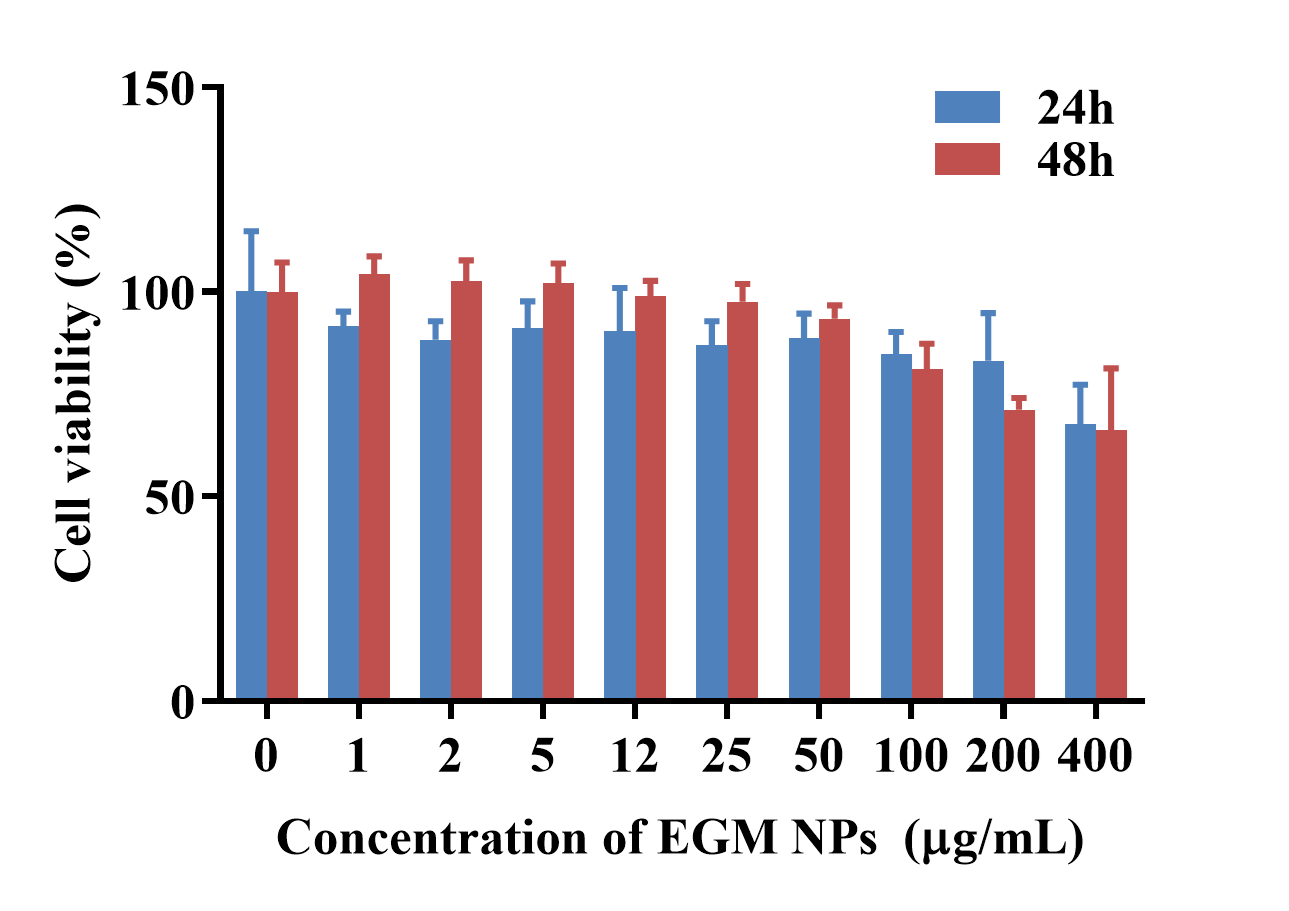

Supplement: Supplementary file 2 [file Image1.png]
